# Combining Chemometric Models with Adsorption Isotherm Measurements to Study Omeprazole in RP-LC

**DOI:** 10.1007/s10337-016-3151-8

**Published:** 2016-08-12

**Authors:** Dennis Åsberg, Marek Leśko, Jörgen Samuelsson, Anders Karlsson, Krzysztof Kaczmarski, Torgny Fornstedt

**Affiliations:** 1 Department of Engineering and Chemical Sciences, Karlstad University, 651 88 Karlstad, Sweden; 2 Department of Chemical and Process Engineering, Rzeszów University of Technology, 35 959 Rzeszow, Poland; 3 AstraZeneca R&D Gothenburg, 431 83 Mölndal, Sweden

**Keywords:** Liquid chromatography, pH, Adsorption isotherm, Design of experiments, Omeprazole

## Abstract

The adsorption of the proton-pump inhibitor omeprazole was investigated using RP-LC with chemometric models combined with adsorption isotherm modelling to study the effect of pH and type of organic modifier (i.e., acetonitrile or methanol). The chemometric approach revealed that omeprazole was tailing with methanol and fronting with acetonitrile along with increased fronting at higher pH. The increased fronting with higher pH for acetonitrile was explored using a pH-dependent adsorption isotherm model that was determined using the inverse method and it agreed well with the experimental data. The model indicated that the peaks exhibit more fronting at high pH due to a larger fraction of charged omeprazole molecules. This model could accurately predict the shape of elution profiles at arbitrary pH levels in the studied interval. Using a two-layer adsorption isotherm model, the difference between acetonitrile and methanol was studied at the lowest pH at which almost all omeprazole molecules are neutral. Omeprazole had adsorbate–adsorbate interactions that were similar in strength for the acetonitrile and methanol mobile phases, while the solute–adsorbent interactions were almost twice as strong with methanol. The difference in the relative strengths of these two interactions likely explains the different peak asymmetries (i.e., tailing/fronting) in methanol and acetonitrile. In conclusion, thermodynamic modelling can complement chemometric modeling in HPLC method development and increase the understanding of the separation.

**Electronic supplementary material:**

The online version of this article (doi:10.1007/s10337-016-3151-8) contains supplementary material, which is available to authorized users.

## Introduction

The pharmaceutical industry quality control (QC) methods, used continuously to release product batches for market, must be validated and then approved by regulatory agencies, such as the US Food and Drug Administration (FDA) [1]. To enable minor post-approval variations to be made to approved QC methods without having to alert regulators, the quality by design (QbD) concept was introduced several years ago [2]. A variant of this approach is a QC-method enhancement concept, where a clear boundary regarding critical attributes is defined together with a principle of the testing technique and an exemplified QC method. In addition to this, an extended robustness testing using the design of experiment (DoE) is required. By doing this, a regulatory flexibility can be given based on the presentation of a deeper scientific knowledge regarding the actual QC method [2, 3]. In QbD, the use of chemometric modeling is encouraged and has become a key strategy [4, 5]. When DoE is used in analytical QbD, the goal is to establish a method operable design region (MODR), where the method performance criteria are met and where variations in responses are understood. Inside the MODR, it is then possible to change experimental conditions without further validation or regulatory interaction [6, 7].

Sometimes other tools, based on firm physicochemical theory are needed to obtain the necessary scientific understanding of a process, especially when considering chromatographic methods. In liquid chromatography, adsorption equilibrium information about a pure component is the most important piece of information for understanding an adsorption process regardless of how many components are present in the system [8]. Recent research has illustrated the importance of investigating adsorption isotherms for understanding the adsorption processes in analytical as well as preparative chromatographic systems [9–13].

Previously, we have studied the proton-pump inhibitor omeprazole [14] in a case study of the method transfer from HPLC to UPLC [13, 15, 16]. The QC method for omeprazole was thoroughly validated in accordance with the ICH guidelines [16]. It was observed, but not reported, that pH was important for the peak shape and retention of omeprazole and that overloaded elution profiles of omeprazole were “anti-Langmuirian”-shaped (type-III [17]) when using acetonitrile as organic modifier. It was also noted that changing the organic modifier to methanol resulted in “Langmuirian”-shaped (type-I [17]) elution profiles. These observations agree with earlier observations that “Langmuirian”-shaped isotherms are found with the use of methanol and S-shaped isotherms that are common with acetonitrile [18]. This was proposed to be because of a multilayer of acetonitrile adsorbing on the surface of the stationary phase compared to the single layer of methanol. To describe the pH dependence, Gritti and Guiochon have developed a model for acidic and basic compounds close to their p*K*_a_ values taking into account their ionization equilibria [19–21]. Changes in the adsorption mechanism due to the nature of the organic modifier and pH are important to consider, but are difficult to study using only the linear part of the adsorption isotherm (using diluted samples). Therefore, we propose also studying the nonlinear part of the adsorption isotherm to get a more complete understanding.

Our aim is to investigating the adsorption of omeprazole as a function of pH and type of organic modifier using a combined DoE and adsorption isotherm approach. We will illustrate how adsorption isotherm modeling can complement chemometric modeling using DoE to obtain a firmer understanding of the separation system.

## Theory

The solute concentrations in the mobile (*C*) and stationary (*q*) phases are related through the adsorption isotherm, and in this work, two adsorption models were used: a two-layer adsorption isotherm model [22] and a model taking the degree of omeprazole ionization into account [19–21], here denoted the pH-dependent model, since the degree of ionization depends on pH. The two-layer adsorption isotherm model assumes that adsorbate–adsorbate interactions occur and can be expressed as [22–24]

1 $$ q = q_{\text{s}} \frac{{b_{\text{S}} C + 2b_{\text{S}} b_{\text{L}} C^{2} }}{{1 + b_{\text{S}} C + b_{\text{S}} b_{\text{L}} C^{2} }}, $$

where *q*_s_ is the saturation capacity, *b*_S_ is the association equilibrium constant at the adsorbent surface, and *b*_L_ is the association equilibrium constant on the first adsorbed layer to the column surface. The two-layer model is an expansion of the Langmuir model incorporating adsorbate–adsorbate interactions between the first established adsorbed solute layer and non-adsorbed solute molecules. Equation (1) is mathematically equivalent to the quadratic [25] and the Moreau [26] adsorption isotherms, but is derived under different assumptions. The adsorption isotherm model that takes omeprazole ionization into account is expressed as [19–21]

2a $$ q = \frac{{\left( {a_{\text{c}} \left( {1 - \alpha } \right) + a_{\text{n}} \alpha } \right)C}}{{1 + \left( {\left( {1 - \alpha } \right)b_{\text{c}} + \alpha b_{\text{n}} } \right)C}}, $$

2b $$ q = \frac{{\left( {a_{\text{c}} \left( {1 - \alpha } \right) + a_{\text{n}} \alpha } \right)C}}{{1 - \left( {\left( {1 - \alpha } \right)b_{\text{c}} + \alpha b_{\text{n}} } \right)C}}, $$

where *a* and *b* are adsorption isotherm parameters. Derived from the general Langmuir model, Eq. (2a) yields Langmuirian peak shapes and Eq. (2b) yields anti-Langmuirian peak shapes [27]. Indices c and n denote the charged and uncharged fractions of omeprazole, respectively, while *α* is the fraction of uncharged omeprazole that is, implicitly, a function of pH. In this study, the mobile phase was weakly buffered and the local pH in the solute band depended on the solute concentration at high loads [19–21].

The set of parameters in the adsorption isotherm model was determined using the inverse method [27] for each experimental condition. In the inverse method, the parameters are estimated by minimizing the sum of squared differences between the experimental and calculated elution profiles [28]. Elution profiles were calculated using the equilibrium-dispersive (ED) model [27], which can be expressed as

3 $$ \frac{\partial C}{\partial t} + F\frac{\partial q}{\partial t} + \frac{u}{{\varepsilon_{t} }}\frac{\partial C}{\partial z} = D_{\text{a}} \frac{{\partial^{2} C}}{{\partial z^{2} }}, $$

where $F = (1 - \varepsilon_{\text{t}} )/\varepsilon_{\text{t}}$ is the phase ratio, *ε*_*t*_ is the total porosity, *u* is the superficial velocity, *D*_a_ is the apparent dispersion coefficient, *t* and *z* are the time and axial positions in the column, respectively, and *C* and *q* are the local mobile and stationary phase solute concentrations, respectively. The orthogonal collocation on the finite-element method [29] was used to discretize the spatial derivatives of the ED model, while the Adams–Moulton method implemented in the VODE procedure [30] was used to solve the system of ordinary differential equations. At *t* = 0, the stationary phase was in equilibrium with the pure mobile phase. Danckwerts boundary conditions were used at the column inlet and outlet [27] with an experimentally obtained injection profile [13]. The minimization was done using a modified least square Marquardt algorithm [31]. Calibration from response to concentration for the experimental elution profiles was done by fitting the different column loads for each experimental condition, so that the injected mass equaled the eluted mass [13].

## Materials and Methods

### Chemicals

Gradient grade acetonitrile (VWR International, Radnor, PA, USA) and HPLC grade methanol (Fischer Scientific, Loughborough, UK) were used as organic modifiers. Water with a conductivity of 18.2 MΩ cm from a Milli-Q Plus 185 water purification system (Merck Millipore, Billerica, MA, USA), analytical grade sodium phosphate dibasic dihydrate, sodium phosphate monobasic dihydrate (Sigma-Aldrich, St. Louis, MO, USA), and ammonium bicarbonate (99 %; J.T. Baker, Deventer, Netherlands) were used to prepare the aqueous buffers. The solutes were omeprazole (>99 %), methyl-omeprazole (analytical reference standard), and omeprazole sulfone (analytical reference standard) and were gifts from AstraZeneca R&D (Mölndal, Sweden). The column hold-up volume was determined by means of pycnometry [32] using acetonitrile and dichloromethane. The aqueous buffers and sample solutions were filtered through a 0.2-μm nylon filter membrane (Whatman, Maidstone, UK) before use.

### Instrumentation

The experiments were performed on an Agilent 1200 chromatograph (Agilent Technologies, Palo Alto, CA, USA) equipped with a binary pump, an auto sampler with a 900-μL sample loop, a diode-array UV-detector, and a thermostated column oven. The extra column volume from the auto sampler to the detector was 0.039 mL and was subtracted from the experimental data. The column was a 100 mm × 4.6 mm XBridge BEH C_18_ column (Waters, Milford, MA, USA) with an average particle diameter of 3.5 µm. Two columns were used, the first in the DoE part and the second in all other experiments, with column hold-up volumes of 1.055 and 0.925 mL, respectively. The flow rates were 1.0 and 0.7 mL min^−1^ for the acetonitrile and methanol mobile phases, respectively. Analytical peaks were detected at 302 nm, while overloaded peaks were detected at 342 nm.

### Procedure

The mobile phases used were either 25/75, v/v, acetonitrile/aqueous buffer or 45/55, v/v, methanol/aqueous buffer. The aqueous buffers were 15 mM phosphate buffer of pH 7.0–9.0 in the DoE part and adsorption isotherm experiments; when estimating the p*K*_a_ values, 15 mM ammonium bicarbonate buffers of pH between 9.0 and 11.0 were used along with an additional phosphate buffer of pH 6.0.

${}_{\text{w}}^{\text{s}} {\text{pH}}$ is pH measured in the eluent containing the organic modifier, i.e., measured directly in the eluent, although the pH-electrode is calibrated with water solutions [33]. To experimentally estimate the ${}_{\text{w}}^{\text{s}} {\text{p}}K_{\text{a}}$ values of omeprazole and omeprazole sulfone, which both behave as monoprotic acids in the investigated pH range, at 30 °C, the retention factor at different ${}_{\text{w}}^{\text{s}} {\text{pH}}$ values were obtained and the results were fitted to the approximate model [34]:

4 $$ k = \frac{{k_{0} + k_{1} \; \times \;10^{{{\text{pH}} - {\text{p}}K_{\text{a}} }} }}{{1 + 10^{{{\text{pH}} - {\text{p}}K_{\text{a}} }} }}, $$

where *k*_0_ and *k*_1_ are the retention factors for the acid and basic forms, respectively. Seven different pH levels were used for the acetonitrile case, and eight different pH levels were used for the methanol case. The retention factor was determined with three replicate measurements at each pH level. Equation (4) neglects changes in the surface properties of the adsorbents, but is sufficiently accurate to estimate the p*K*_a_ values of specific solvent mixtures and temperatures in the ${}_{\text{w}}^{\text{s}} {\text{pH}}$ range applicable here.

Column temperature (20–40 °C) and pH (${}_{\text{w}}^{\text{w}} {\text{pH}}$ 7.0–9.0) were chosen as factors in the experimental design, which was a full factorial design in three levels with three center points. For each run, 10-μL samples containing 0.15 mg mL^−1^ omeprazole, 0.011 mg mL^−1^ omeprazole sulfone, and 0.007 mg mL^−1^ methyl-omeprazole were injected in duplicate. The diluent was the corresponding mobile phase for each run. As responses, the retention and resolution factors for all components and the tailing factor for omeprazole were used. Regression models were constructed in the software MODDE 7 (Umetrics, Sweden) after first removing outliers and insignificant coefficients at a 95 % confidence level.

To determine the adsorption isotherm of omeprazole at different pH levels, overloaded, duplicate injections of 300, 400, and 500 μL were made at five pH levels in the same range as used in the DoE. The column temperature was 30 °C, and the experiments were performed with either acetonitrile or methanol as organic modifier. The omeprazole concentration was 2.5 mg mL^−1^ with the acetonitrile mobile phase and 4.0 mg mL^−1^ with the methanol mobile phase, while the diluent was the mobile phase.

## Results and Discussion

### Chemometric Modeling

**Fig. 1:**
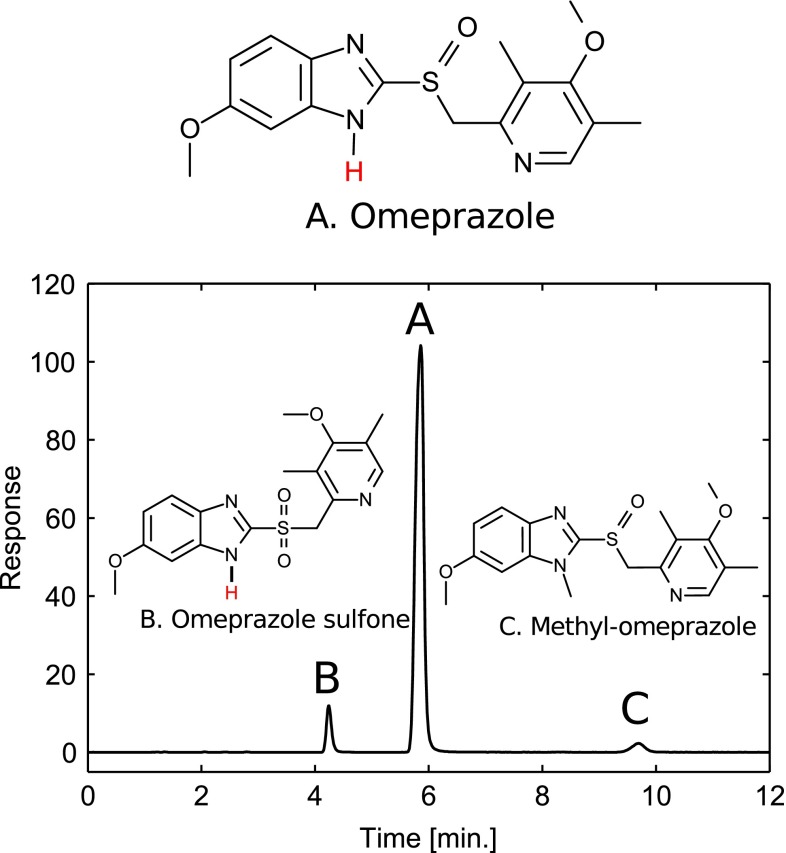
Structure of the omeprazole and the investigated impurities along with the chromatogram obtained at the center point (buffer ${}_{\text{w}}^{\text{w}} {\text{pH}}$ = 8.0, 30 °C) of the experimental design with acetonitrile as organic modifier. The flow rate was 1.0 mL min^−1^, the detection was conducted at 302 nm, and the injection was 10 μL of 0.15 mg mL^−1^ omeprazole, 0.011 mg mL^−1^ omeprazole sulfone, and 0.007 mg mL^−1^ methyl-omeprazole. The hydrogen of the benzimidazole group lost at high pH for omeprazole and omeprazole sulfone is indicated in red

**Fig. 2:**
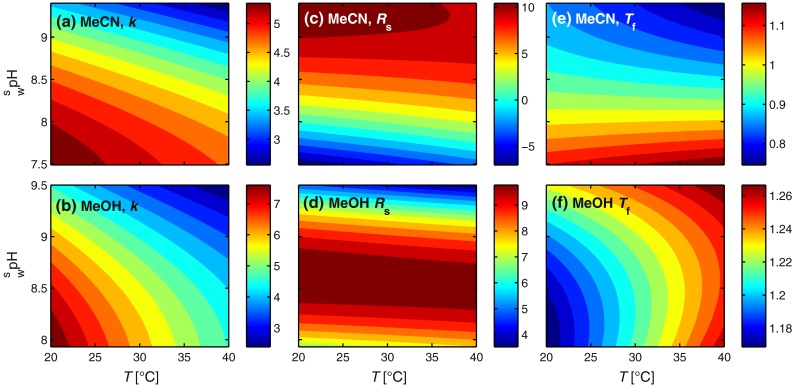
Response surfaces from the experimental designs with acetonitrile (**a**, **c**, **e**) and methanol (**b**, **d**, **f**) as organic modifier. **a**, **b** Retention factors, *k*, of omeprazole, **c**, **d** resolution factors, *R*_s_, between omeprazole and H168/66, and **e**, **f** tailing factors, *T*_f_, of omeprazole

**Fig. 3:**
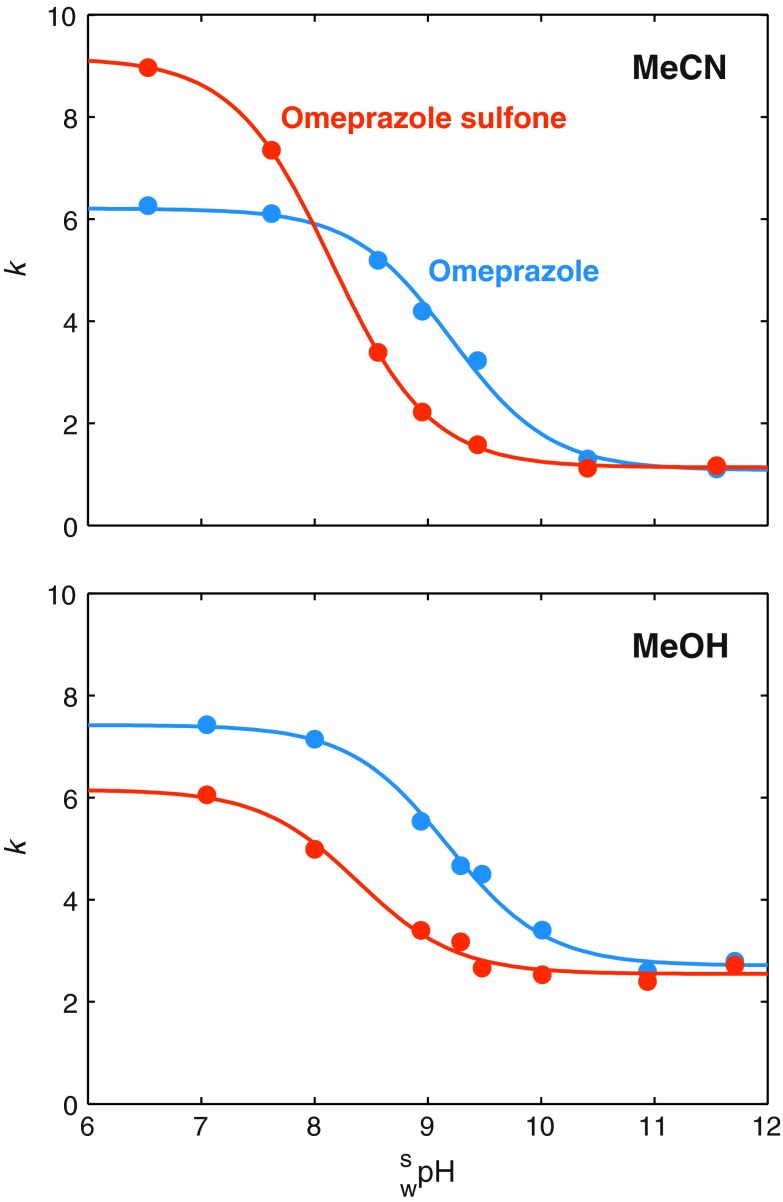
Estimation of p*K*_a_ values for omeprazole and omeprazole sulfone in 25/75, v/v, acetonitrile/water and 45/55, v/v methanol/water at 30 °C. *Symbols* are experimental retention factors at different mobile phase ${}_{\text{w}}^{\text{s}} {\text{pH}}$ levels and *solid lines* are the best fit to Eq. (4)

The design region was chosen to span common HPLC temperatures and a relevant pH range for the separation system (omeprazole quickly degrades below 7 [35]). From the full factorial design, excellent regression models could be determined for all responses. The regression coefficients and statistics are presented in Electronic Supplementary Material Tables S1 and S2. The structures of the solutes and the chromatogram of the center point with acetonitrile are shown in Fig. 1. From the regression models, response surfaces were constructed for each response. Figure 2a, b shows the retention factors for omeprazole with acetonitrile and methanol as organic modifiers, respectively, with pH being the most important factor. The same trends are present for acetonitrile and methanol, i.e., increasing pH and temperature reduces the retention factor, but temperature has a comparatively larger effect with methanol. Omeprazole sulfone behaves similarly to omeprazole (Electronic Supplementary Material Fig. S1), i.e., the retentions are decreasing with increasing temperature and pH. For omeprazole and omeprazole sulfone, the retention factor decreases with increasing pH, because the hydrogen on the benzimidazole group is lost at high pH (Fig. 1) and the molecules go from being neutral to being negatively charged. Since the stationary phase is apolar and the mobile-phase polar in RP-LC, charged compounds are less retained than neutral ones. The p*K*_a_ values for omeprazole and omeprazole sulfone in the mobile phase at 30 °C were determined experimentally by measuring the retention factors with three replicates at different pH values and fitting the data to Eq. (4), and the results are shown in Fig. 3. The p*K*_a_ values, given with 95 % confidence intervals, for omeprazole were 9.21 ± 0.16 and 9.18 ± 0.14 with acetonitrile and methanol, respectively. For omeprazole sulfone, the p*K*_a_ values were 8.15 ± 0.03 and 8.38 ± 0.29 with acetonitrile and methanol, respectively. The temperature dependence of the p*K*_a_ value for the phosphate buffer is −0.0028 units/K [36] and can be neglected in the studied temperature interval. For secondary amines, it is around −0.01 units/K [37], giving a change of ±0.1 units in the design region due to temperature. For methyl-omeprazole, the retention factor is almost unaffected by pH, since the acidic hydrogen on the benzimidazole group is replaced with a methyl group.

Figure 2c, d shows response surfaces for the resolution factor between omeprazole and omeprazole sulfone. For acetonitrile, the elution order is changed with the peaks co-eluting around ${}_{\text{w}}^{\text{s}} {\text{pH}}$ = 7.8, while for methanol, omeprazole always elutes after omeprazole sulfone with a maximum resolution factor at around ${}_{\text{w}}^{\text{s}} {\text{pH}}$ 8.5. In both the cases, pH is the most important factor with temperature playing a minor role. The difference in p*K*_a_ can quantitatively account for the reversal of peak order observed in Fig. 2c (cf. Fig. 3). The resolution factors between omeprazole and methyl-omeprazole versus pH and temperature are shown in Electronic Supplementary Material Fig. S2. For acetonitrile, the temperature is not significant, leading to a one-factor model in which the resolution factor increases with pH, but is never below nine. With methanol, on the other hand, omeprazole and methyl-omeprazole nearly co-elutes at the lowest pH and temperature indicating a change in selectivity when switching modifier. One reason for this is that acetonitrile forms a double layer on the stationary phase surface, while methanol forms a monolayer [18]. The differences between acetonitrile and methanol will be discussed further in “Adsorption isotherm modeling”.

**Fig. 4:**
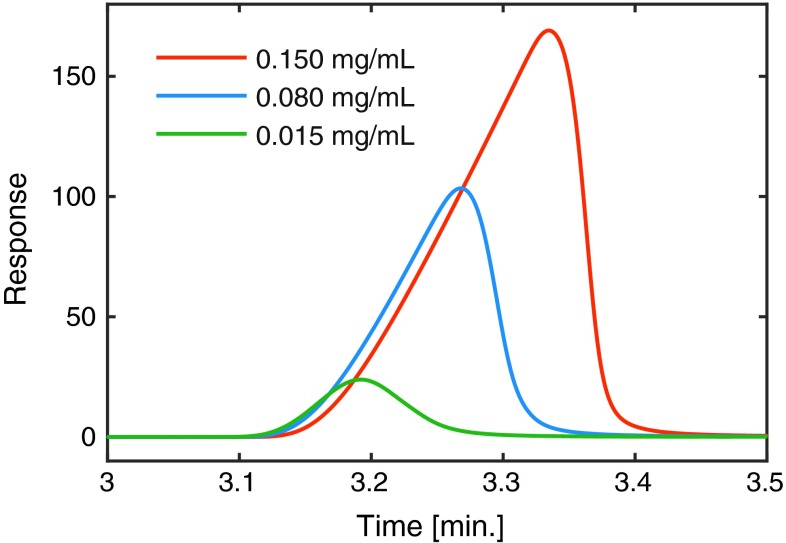
Elution profiles of omeprazole using different sample concentrations to illustrate that the fronting is due to thermodynamic overloading. The mobile phase with 25/75, v/v, acetonitrile/phosphate buffer (${}_{\text{w}}^{\text{w}} {\text{pH}}$ = 9.0) at a temperature of 30 °C and flow rate of 1.0 mL/min

Response surfaces for the peak tailing, calculated according to the USP definition, are shown in Fig. 2e and f. With acetonitrile as modifier, the peak is tailing at low pH and fronting (i.e., a tailing factor below one) at high pH with the temperature only playing a minor role. Methanol, in contrast, gives only tailing peaks (i.e., tailing factor above one) with temperature being the most important factor. This difference cannot be directly explained from the observations in the DoE investigation. By injecting samples of different concentrations and observings if the peak shape changes, it is possible to get an indication if the origin of the peak asymmetry is thermodynamic or kinetic. The results are presented in Fig. 4, where three concentrations of omeprazole are injected at ${}_{\text{w}}^{\text{s}} {\text{pH}}$ 9.38 and 40 °C. The peaks clearly become more symmetrical when the concentration is decreasing; therefore, the underlying reason for the peak asymmetry is likely of thermodynamic in nature [27]. Note that in QC methods, it is often necessary to have concentrations in the 0.1 mg/mL range of the active pharmaceutical ingredient to obtain sufficiently high signals for the impurities [38, 39].

### Adsorption Isotherm Modeling

This section seeks to explain the reason for the peak asymmetry observed in Fig. 2e and f due to pH. To do this, the adsorption isotherms for omeprazole, with methanol and acetonitrile as organic modifiers, were determined directly from overloaded elution profiles using the inverse method. Experimental overloaded elution profiles are shown in Fig. 6 (blue lines). The profile with acetonitrile as modifier is “anti-Langmuirian” in shape having a diffuse front and a sharp rear, and at ${}_{\text{w}}^{\text{s}} {\text{pH}}$ values above 9, the diffuse front of the elution profile is increasingly curved. With methanol as modifier, at ${}_{\text{w}}^{\text{s}} {\text{pH}}$ values up to approximately 9, the elution profiles are “Langmuirian” in shape having a sharp front and a diffuse rear.

When determining adsorption isotherms using the inverse method, one must properly select an adsorption isotherm model a priori. One important characteristic of the adsorption isotherm is the number of adsorption sites, usually determined from the adsorption energy distribution (AED) obtained from experimental data using, for example, the frontal analysis (FA) [27]. The AED for omeprazole, using the same stationary and mobile phases (${}_{\text{w}}^{\text{s}} {\text{pH}} \approx 8. 5$) as in this work, was previously determined from FA [13] and found to be unimodal, i.e., containing only one type of adsorption site. Since the DoE investigation revealed that the retention factor was strongly pH dependent and that the p*K*_a_ value of omeprazole was inside the studied pH range, the adsorption model should take pH into account. Furthermore, when injecting large amounts of omeprazole, the buffer had insufficient capacity to keep the pH constant, so the pH will depend on the local omeprazole concentration.

**Fig. 5:**
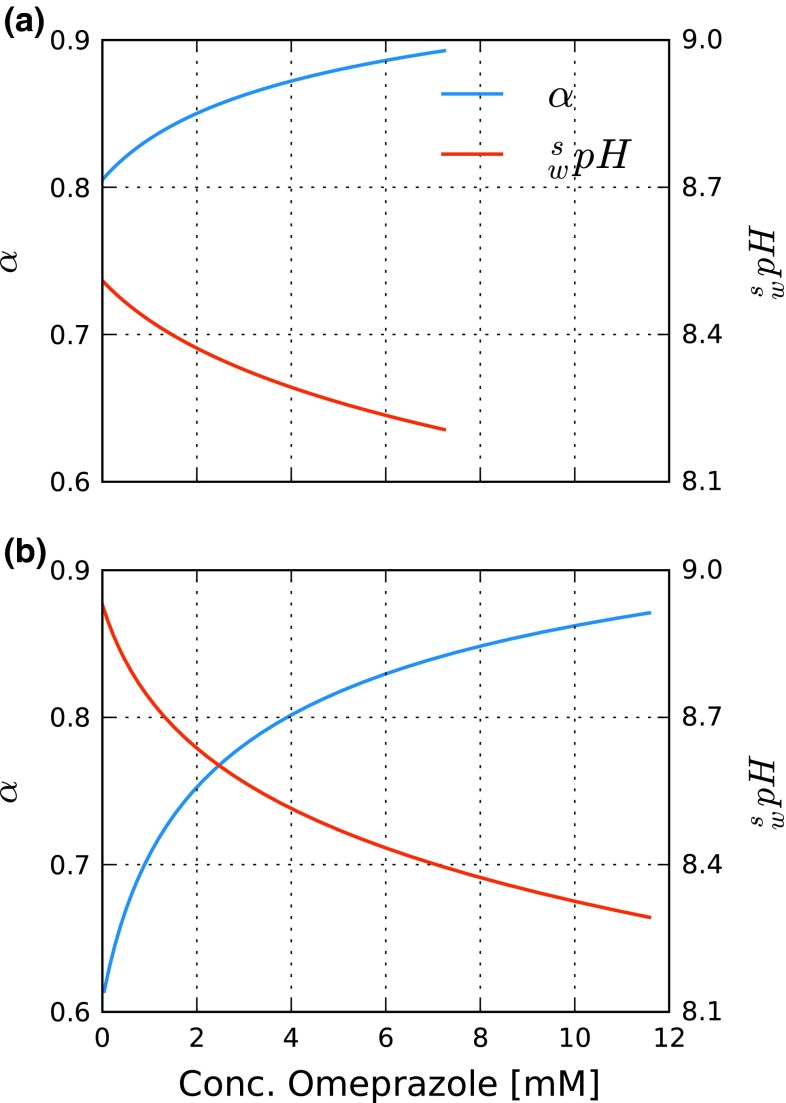
Fraction of the neutral form of omeprazole, *α*, and ${}_{\text{w}}^{\text{s}} {\text{pH}}$ versus the concentration of omeprazole in the mobile phase with **a** 25/75, v/v, acetonitrile/phosphate buffer (${}_{\text{w}}^{\text{w}} {\text{pH}}$ = 9.0) and **b** 45/55, v/v, methanol/phosphate buffer (${}_{\text{w}}^{\text{w}} {\text{pH}}$ = 9.0)

**Fig. 6:**
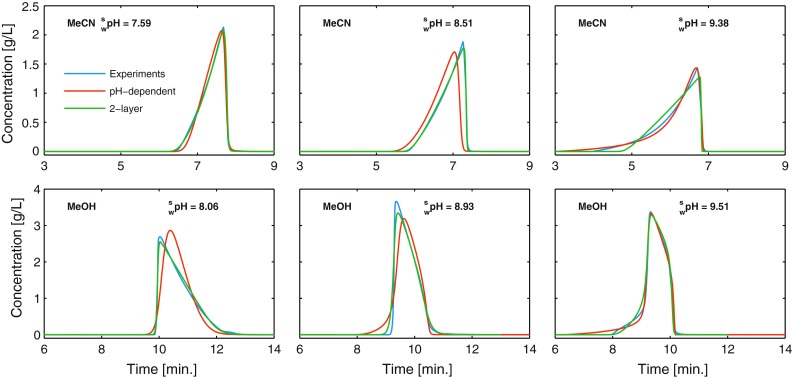
Comparison between experimental elution profiles (*blue lines*) and elution profiles calculated using the pH-dependent isotherm model (*red lines*) and the quadratic isotherm model (*green lines*) for omeprazole at different pH levels. In **a**–**c** 25/75, v/v, acetonitrile/phosphate buffer is used as mobile phase, and in **d**–**f** 45/55, v/v, methanol/phosphate buffer is used as mobile phase. For acetonitrile, the flow rate was 1.0 mL min^−1^ and injections were 0.5 mL of a 2.5 g L^−1^ solution. For methanol, the flow rate was 0.7 mL min^−1^ and injections were 0.5 mL of a 4.0 g L^−1^ solution. The column temperature was 30 °C in all experiments. Note that for the pH-dependent model, one set of numerical parameters is used at all pH levels, while for the quadratic model, a different parameter set is used at each pH

One adsorption isotherm model accounting for this situation is the pH-dependent model, Eq. (2), derived by Gritti and Guiochon [19–21]. Rather, lengthy calculations are needed to determine the function *α*(*C*), i.e., the fraction of uncharged omeprazole molecules as a function of the total omeprazole concentration. Using activity coefficients and the ${}_{\text{w}}^{\text{s}} {\text{p}}K_{\text{a}}$ derived in the previous section, the results for the highest pH cases are shown in Fig. 5 and the calculations are described in detail in the Electronic Supplementary Material. The pH-dependent isotherm model, Eq. (2), has the advantage that only one set of parameters is needed for modeling at arbitrary pH (i.e., in the inverse method) and only elution profiles at the highest and lowest pH levels being needed. The estimated sets of parameters for acetonitrile, Eq. (2b), and methanol, Eq. (2a), are presented in Electronic Supplementary Material Table S3 along with some details of the estimation procedure. The agreement between calculated and experimental elution profiles was good in both methanol and acetonitrile (see red lines in Fig. 6). Note that the intermediate pH in Fig. 6 was not used in the inverse method; the profiles at this level are predictions and, therefore, agree somewhat less with the experimental elution profiles. That the pH-dependent isotherm was able to describe the experimental elution profiles well lends strength to the proposed mechanism that the relationship between the charged and uncharged forms of omeprazole causes the increased fronting at high pH. When the charged form increases at high pH, the elution profiles become more deformed, with parts of the front starting to move faster than the rest of the profile. We concluded that the increased fronting of the omeprazole peaks with increased pH seen in the DoE investigation with acetonitrile as modifier can be explained by thermodynamic overloading combined with variation in the local eluent pH due to the weakly buffered mobile phase.

To study the difference between methanol and acetonitrile, we consider the case in which most (>95 %) omeprazole molecules are uncharged, i.e., at the lowest pH (buffer pH 7.0). The two-layer isotherm model, Eq. (1), is fitted to each pH separately to obtain individual sets of parameters. At higher pH where the fraction of uncharged omeprazole decreases, the two-layer isotherm should only be seen as an empirical model, since it does not take into account the charge of omeprazole or the variation in the local eluent pH due to the weakly buffered mobile phase. However, at the lowest pH, it can yield certain physiochemical insights. The adsorption isotherm parameters for the two-layer isotherm is presented in Electronic Supplementary Material Table S4, and compared with the parameters at the lowest pH for methanol and acetonitrile, the following is observed: (1) the saturation capacity is higher for acetonitrile, (2) the equilibrium constant for the adsorbent, *b*_s_, is almost twice as large for methanol, and (3) the equilibrium constant for the adsorbed solute layer, *b*_L_, is almost equal to that for methanol. Previously, it has been shown that acetonitrile adsorbs in multilayers to the C_18_ chains, while methanol adsorbs in a monolayer [18, 40]. The thicker acetonitrile layer can dissolve more solute molecules from the bulk than can the bonded C_18_ layer alone, which could be the reason for the higher saturation capacity. The acetonitrile multilayer could also explain the lower *b*_s_ with acetonitrile, since omeprazole molecules have more difficulty interacting with the C_18_ chains due to the thicker acetonitrile layer. From the above speculation, one could expect that omeprazole has a similar *b*_L_ with acetonitrile and with methanol, since this reflects the interaction between omeprazole molecules surrounded by the mobile phase, which consists mainly of water in both the cases. The magnitude of *b*_s_ relative to *b*_L_ with the two modifiers indicates that adsorbate–adsorbate interactions are more favored in acetonitrile than in methanol, which is believed to be the main reason for the change in peak shape for uncharged omeprazole when switching organic modifier.

## Conclusions

The adsorption of omeprazole as a function of pH for two organic modifiers, acetonitrile and methanol, has been investigated through adsorption isotherm characterization. The aim was to determine an adsorption isotherm model for the adsorption of omeprazole to demonstrate how such knowledge could provide complementary information to support the chemometric modeling commonly used in the QbD framework. The system considered here contained omeprazole along with two of its impurities, and the buffer pH and temperature were varied in a DoE investigation.

From the DoE results, it appeared that the selectivity differed between acetonitrile and methanol as modifiers, omeprazole having the critical resolution factor with omeprazole sulfone in acetonitrile and with methyl-omeprazole in methanol. This was explained partly by the differences in the mobile phase pH and p*K*_a_ values of the solutes in the acetonitrile and methanol mobile phases. Furthermore, the DoE results also revealed that omeprazole was tailing with methanol and fronting with acetonitrile, along with increased fronting at high pH. These observations could not be explained by the DoE results, so after confirming that the underlying origin of the asymmetry was thermodynamic, the adsorption isotherms were determined to deepen the understanding.

The increase in fronting with pH in the acetonitrile case was understood by fitting a pH-dependent adsorption isotherm simultaneously to all mobile-phase pH values. This model contains the fractions of neutral omeprazole molecules, which are, implicitly, a function of the local mobile-phase pH in the solute band. The pH-dependent model agreed well with the experimental data and indicated that the peaks exhibit more fronting at high pH due to a larger fraction of charged omeprazole molecules. This model could also accurately predict overloaded elution profiles at arbitrary pH in the studied interval.

The difference between acetonitrile and methanol was studied at the lowest pH at which almost all omeprazole molecules are in the neutral state, using a two-layer adsorption isotherm model. From the determined adsorption isotherms, we found that (1) the saturation capacity was larger with acetonitrile, (2) the association equilibrium constant for adsorbate-adsorbent interactions is about a factor two higher with methanol, and (3) the association equilibrium constant for adsorbate–adsorbate interactions is similar for the two organic modifiers. Points (1) and (2) were believed to be due to the adsorbed multilayers of acetonitrile making it possible to dissolve more solute molecules from the bulk than could the bonded layer alone making it more difficult for omeprazole molecules to interact with the C_18_ chains due to the thickness of the acetonitrile layer. The acetonitrile multilayer lowered the solute-adsorbent equilibrium constant, since omeprazole molecules have more difficulty interacting with the C_18_ chains due to the thick acetonitrile layer. The difference in relative strength between the two equilibrium constants for the two modifiers is believed to cause the “Langmuir”/”anti-Langmuir” difference.

We strongly believe that thermodynamic modeling can be a useful tool to complement chemometric models for the HPLC method validation in the QbD framework. Additional scientific-based information beside the DoE investigation is of high importance to present and find acceptance for an enhanced QC-method concept. Depths in scientific knowledge make it possible for the regulatory agencies to give the pharmaceutical industry an increased flexibility that allow continuous improvement of regulatory approved QC methods. Thereby, a high-quality release process of product batches can be maintained during the whole life cycle of the product. An improved understanding of the separation process and the ability to predict the shape of overloaded elution profiles can be achieved at the cost of only a few more experiments.

## Electronic supplementary material

Below is the link to the electronic supplementary material.

Supplementary material 1 (DOCX 391 kb)
