# Supplementary material for: Combining Chemometric Models with Adsorption Isotherm Measurements to Study Omeprazole in RP-LC
Source: Chromatographia. 2016 Aug 12;79(19):1283–91. doi: 10.1007/s10337-016-3151-8 (PMC5039227; doi:10.1007/s10337-016-3151-8)
Supplement: Supplementary file 1 — Supplementary material 1 (DOCX 391 kb) [file 10337_2016_3151_MOESM1_ESM.docx]

Combining Chemometric Models with Adsorption Isotherm Measurements to Study Omeprazole in RP-LC

Dennis Åsberg^1^, Marek Leśko^2^, Jörgen Samuelsson^1*^, Anders Karlsson^3^, Krzysztof Kaczmarski^2*^ and Torgny Fornstedt^1^

^1^Department of Engineering and Chemical Sciences, Karlstad University, SE-651 88 Karlstad, Sweden

^2^Department of Chemical and Process Engineering, Rzeszów University of Technology, PL-35 959 Rzeszów, Poland

^3^AstraZeneca R & D, SE-431 83 Mölndal, Sweden

*Corresponding authors’ information:

J. Samuelsson: phone: +46 54 700 1620; fax: +46 54 700 2040; email: Jorgen.Samuelsson@kau.se.

K. Kaczmarski: phone: +48 17 865 1295; fax: +48 17 854 3655; email: kkaczmarski@prz.edu.pl.

Supplementary Data

## Design of Experiments

Additional results obtained from the regression models derived from the design of experiments investigation are presented below. In Table S1 and S2 the regression coefficients are listed along with the quality of the fit in terms of variation explained by the model (*R*^2^_adj_) and variation predicted by the model (*Q*^2^). The models were obtained after excluding outliers and insignificant coefficients using a 95% confidence level. In Fig. S1, the response surfaces for the retention factor of the impurities are shown. Note the larger effect of temperature seen when methanol is used as organic modifier, which is also reflected in the absolute value of the regression coefficients for temperature seen in Table S1 and S2. Fig. S3 shows the resolution factor of omeprazole and H193/61 which is much larger for acetonitrile than for methanol.

**Table S1:** Regression coefficients for the DoE in the *T-*pH space with acetonitrile as organic modifier. *k* is the retention factor, *R*_s_ is the resolution factor and *T*_f_ is the tailing factor. The value 0 denotes that the coefficient was statistically insignificant at 95% confidence level.

| Coefficient | *k*_OM_ | *k*_H168/66_ | *k*_H193/61_ | *R*_s, H168/66-OM_ | *R*_s, H193/61-OM_ | *T*_f, OM_ |
| --- | --- | --- | --- | --- | --- | --- |
| Constant | -17.78 | 106.5 | 14.63 | -419.9 | 173.1 | 4.732 |
| *T* | 0.02814 | -0.4303 | -0.03369 | 1.420 | 0 | 0.04026 |
| pH | 6.471 | -20.11 | -1.224 | 87.58 | -42.64 | -0.8536 |
| *T***T* | 0 | 0.0006125 | -0.0002414 | 0 | 0 | 0 |
| pH*pH | -0.4355 | 0.9667 | 0.07127 | -4.413 | 2.773 | 0.04857 |
| *T**pH | -0.008330 | 0.03888 | 0 | -0.1601 | 0 | -0.004959 |
| *R*^2^_adj_ | 1.000 | 1.000 | 0.999 | 0.999 | 0.992 | 0.966 |
| *Q*^2^ | 0.999 | 0.998 | 0.997 | 0.997 | 0.987 | 0.911 |

**Table S2:** Regression coefficients for the DoE in the *T-*pH space with methanol as organic modifier. *k* is the retention factor, *R*_s_ is the resolution factor and *T*_f_ is the tailing factor. The value 0 denotes that the coefficient was statistically insignificant at 95% confidence level.

| Coefficient | *k*_OM_ | *k*_H168/66_ | *k*_H193/61_ | *R*_s, H168/66-OM_ | *R*_s, H193/61-OM_ | *T*_f, OM_ |
| --- | --- | --- | --- | --- | --- | --- |
| Constant | -28.78 | 60.62 | 7.995 | -505.0 | 310.3 | 2.453 |
| *T* | -0.4844 | -0.5251 | -0.2506 | 0.6262 | -0.1804 | 0.01844 |
| pH | 11.77 | -9.952 | 1.125 | 118.1 | -77.42 | -0.3815 |
| *T***T* | 0.001631 | 0.001971 | 0.001893 | 0 | 0 | 0 |
| pH*pH | -0.8273 | 0.4507 | -0.07346 | -6.763 | 4.796 | 0.02616 |
| *T**pH | 0.02838 | 0.03478 | 0 | -0.07388 | 0.03575 | -0.001719 |
| *R*^2^_adj_ | 0.999 | 1.000 | 1.000 | 0.998 | 0.999 | 0.894 |
| *Q*^2^ | 0.992 | 0.999 | 1.000 | 0.994 | 0.998 | 0.837 |


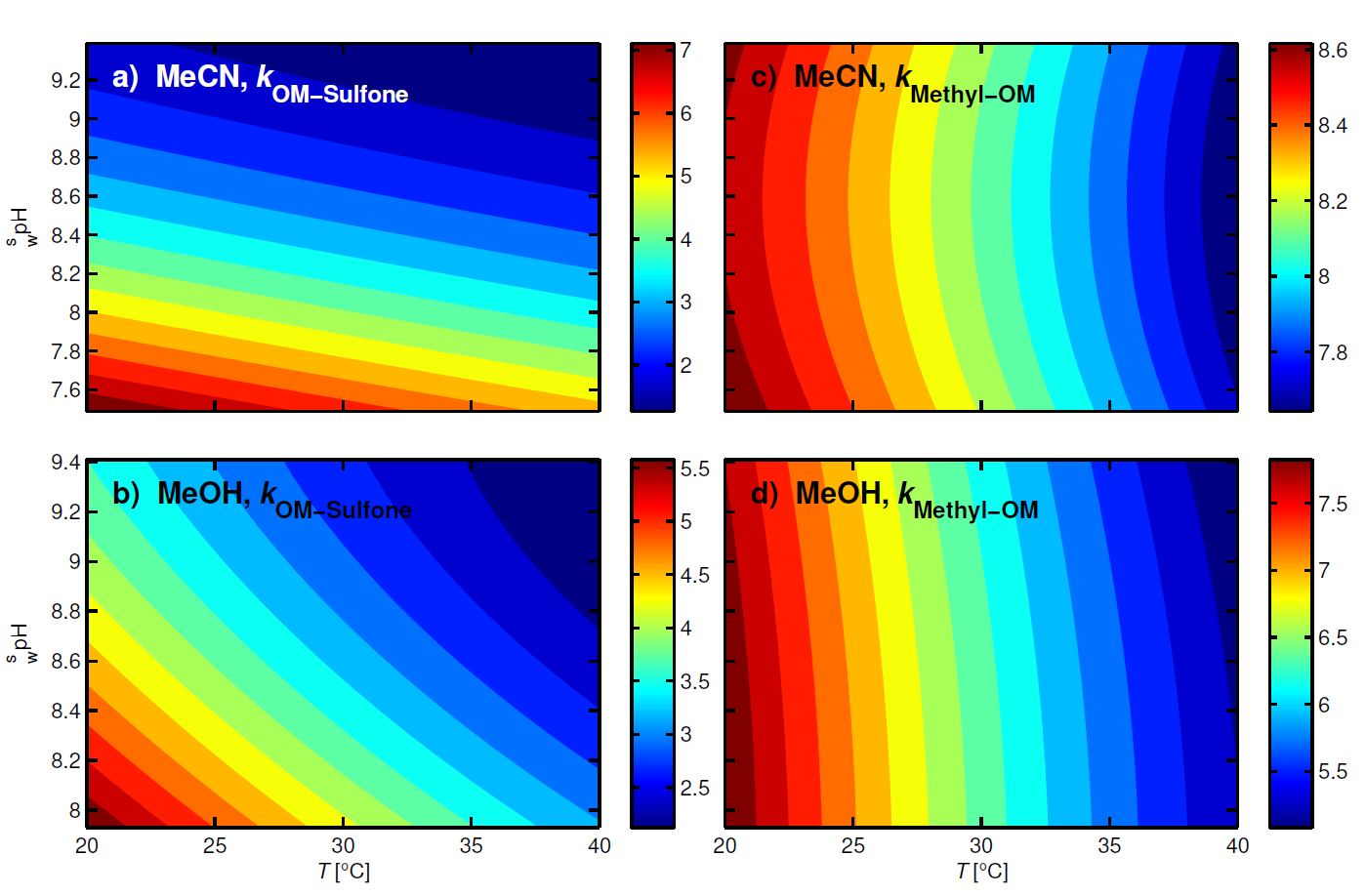


**Fig. S1:** Response surfaces from the experimental design for the retention factor of the impurities. a) and b) are H168/66 with acetonitrile and methanol as organic modifier, respectively. c) and d) is H193/61 with acetonitrile and methanol respectively. Note that H193/61 only has a very slight dependence on pH.


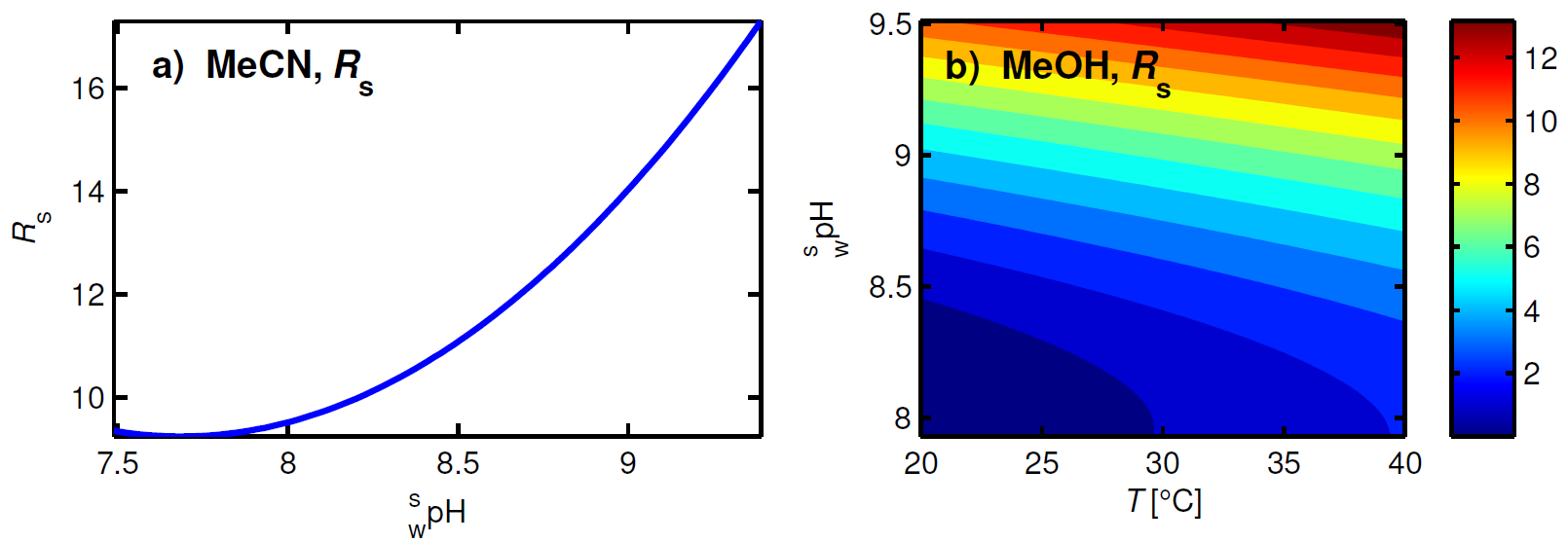


**Fig. S2:** The regression models for the resolution factor between omeprazole and H193/61 for a) acetonitrile as the organic modifier where temperature as a factor is statistically insignificant and b) methanol. Note the large resolution found at all pH with acetonitrile and that the peaks nearly co-elute at low pH and temperature with methanol.

## pH Calculations

In this study, samples were prepared by dissolving the neutral form of omeprazole in the eluent. The fraction of uncharged omeprazole in the sample depends both on the eluent and the sample concentration. To calculate this, two equilibria are considered:

$$H_{2}PO_{4}^{-}\rightleftharpoons HPO_{4}^{2-}+H^{+}$$

$$HA\rightleftharpoons A^{-}+H^{+}$$

where HA is omeprazole in its neutral form and A^-^ is the charged form. Other phosphate, omeprazole, water and methanol equilibria are omitted based on Fig. S4.


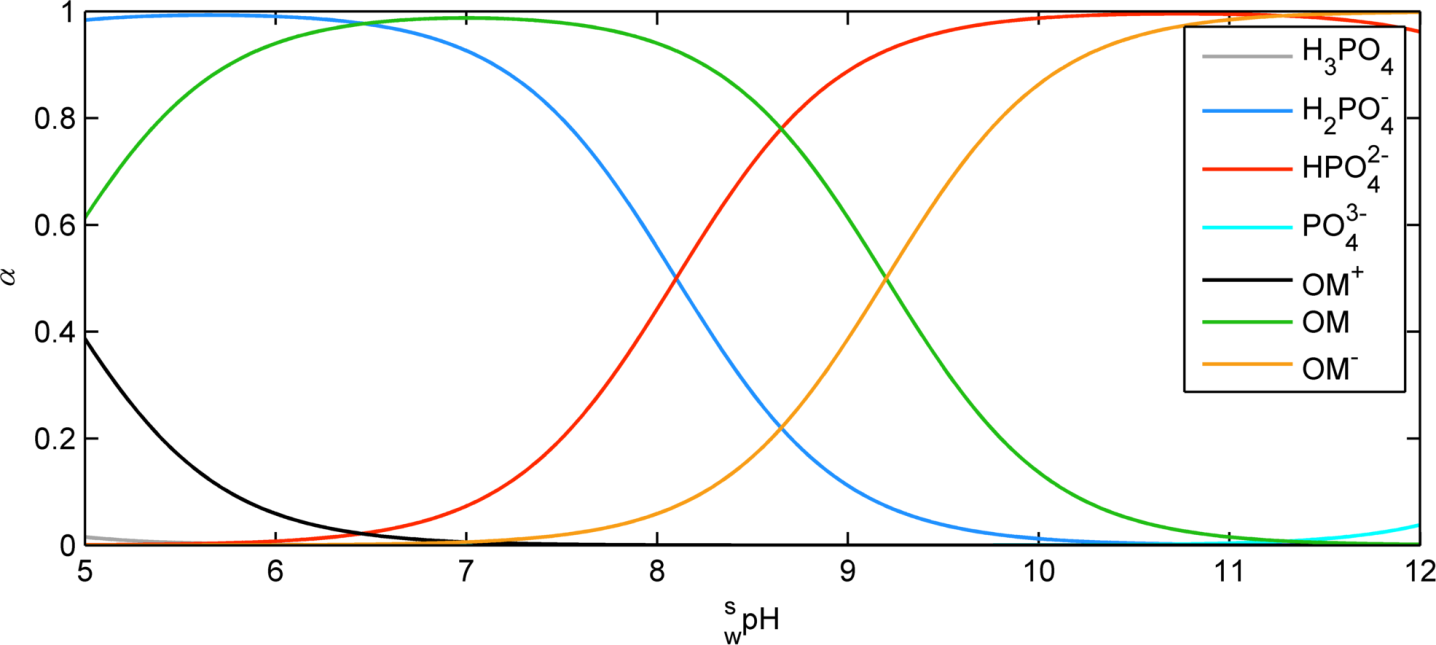


**Fig. S3:** Approximate fraction of different protolytic species (*α*) in the mobile phase (MeOH) as a function of pH. The dashed lines are the relevant area for the experiments.

This problem was solved by combining the two equilibria and assuming a change of *x* according to:

|  | $\mathbf{HPO}_{\mathbf{4}}^{\boldsymbol{2-}}$ | $\mathbf{HA}$ | $\boldsymbol{\rightleftharpoons}$ | $\mathbf{H}_{\mathbf{2}}\mathbf{P}\mathbf{O}_{\mathbf{4}}^{\mathbf{-}}$ | $\mathbf{A}^{\mathbf{-}}$ |
| --- | --- | --- | --- | --- | --- |
| Start | $A$ | $D$ | $\rightleftharpoons$ | $B$ | $E$ |
| Change | $-x$ | $-x$ | $\rightleftharpoons$ | $+x$ | $+x$ |
| Final stage | $A-x$ | $D-x$ | $\rightleftharpoons$ | $B+x$ | $E+x$ |

which gave the equilibrium expression:

| $\frac{K_{a}}{K_{s}}=\frac{\gamma_{B}\left( B+x \right)\gamma_{E}(E+x)}{\gamma_{A}\left( A-x \right)\gamma_{D}(D-x)}$ | (S1) |
| --- | --- |

where *γ* is the activity coefficient. The dissolved omeprazole used in this study was uncharged so *E* = 0. *D* represents the uncharged form of omeprazole so *γ_D_* = 1 which gives the quadratic equation when solved for *x*:

| $x^{2}+\frac{kB+A+D}{k-1}x-\frac{AD}{k-1}=0, k=\frac{\gamma_{B}\gamma_{E}}{\gamma_{A}}\frac{K_{a}}{K_{s}}$ | (S2) |
| --- | --- |

The pH, *A* and *B* were estimated using the Henderson-Hasselbalch equation:

| $\mathrm{pH}=pK_{a}+\log_{10}\left( \frac{\gamma_{B}B}{\gamma_{A}A} \right)$ | (S3) |
| --- | --- |

The p*K*_a_-value for the solute and the buffer component is also affected by addition of organic modifier. In this study the p*K*_a_-value for omeprazole were chromatographically estimated to 9.18 in the eluent with 45% (v/v) MeOH and 9.21 in the eluent with 25% (v/v) MeCN. Phosphate p*K*a-values were taken from literature [1, 2].

The activity coefficients were estimated using the extended Debye-Hückel equation:

| $\log\gamma_{i}=\frac{-A_{DH}z^{2}\sqrt{I}}{1+a_{i}B_{DH}\sqrt{I}}$ | (S4) |
| --- | --- |

where *a* is the Debye radius and *I* the ionic strength. The ionic strength was solved iteratively and is defined as:

| $I=0.5\sum z_{i}^{2}C_{i}=0.5\left( \left[ \mathrm{Na}^{+} \right]+\left[ H_{2}\mathrm{PO}_{4}^{-} \right]+4\left[ H\mathrm{PO}_{4}^{2-} \right]+\left[ A^{-} \right]+\left[ {H_{3}O}^{+} \right]+[\mathrm{OH}^{-}] \right)$ | (S5) |
| --- | --- |

The Debye radius was taken from literature [3] except for charged omeprazole where the Bates-Guggenheim convention, which considers a Debye radius of 4.56 Å, was used [4]. *A_DH_* and *B_DH_* are constants and can be calculated as [2]:

| $A_{DH}=\frac{1.8246\cdot{10}^{6}}{\left( \varepsilon T \right)^{3/2}}, B_{DH}=\frac{50.29}{\sqrt{\varepsilon T}}$ | (S6) |
| --- | --- |

Where *ε* is the dielectric constant and *T* is the absolute temperature. The dielectric constants for methanol water mixtures [5] and acetonitrile water mixtures [4] were taken from literature.

## Adsorption Isotherm Parameters

The estimation was been done on the basis of two peaks: buffer pH 9.0, *V*_inj_ = 500 µL and pH 7.0, *V*_inj_ = 300 µL.

The effective (apparent) dispersion coefficient is a function of pH. More precisely the effective diffusion coefficient (inside adsorbent) should be a function of pH, because the degree of dissociation depends on pH. The ion and neutral components have different diffusivity inside adsorbent.

Therefore apart from the isotherm model parameters the apparent dispersion coefficients (only for the pH at which peaks were taken for the estimation) has also been estimated. For other pH the apparent dispersion coefficient was calculated using linear relationship. The result from the inverse method is presented in Table S3.

**Table S3:** Estimated adsorption isotherm parameters for the pH-dependent model with acetonitrile, Eq. (2b) and methanol, Eq. (2a), as organic modifiers.

| Modifier | *a*_n_  [-] | *b*_n_  [L/g] | *a*_c_  [-] | *b*_c_  [L/g] | *D*_a_ pH 7  [cm^2^/min] | *D*_a_ pH 9  [cm^2^/min] |
| --- | --- | --- | --- | --- | --- | --- |
| Methanol | 10.2 | 0.00574 | 3.44 | 0.158 | 0.0290 | 0.0141 |
| Acetonitrile | 9.16 | 0.0265 | 0.0380 | 0.0104 | 0.0195 | 0.0689 |

**Table S4:** Estimated adsorption isotherm parameters for the two-layer isotherm for omeprazole at different pH with acetonitrile and methanol as organic modifiers.

| Parameter | Acetonitrile | | | | |  | Methanol | | | | |
| --- | --- | --- | --- | --- | --- | --- | --- | --- | --- | --- | --- |
| ^s^_w_pH | 7.59 | 8.08 | 8.51 | 9.02 | 9.38 |  | 8.06 | 8.56 | 8.93 | 9.32 | 9.51 |
| *q*_s_ [g/L] | 119 | 135 | 173 | 151 | 189 |  | 76.1 | 92.5 | 93.8 | 93.6 | 57.7 |
| *b*_S_ [L/g] | 0.0720 | 0.0615 | 0.0441 | 0.0422 | 0.0314 |  | 0.133 | 0.101 | 0.0906 | 0.0855 | 0.128 |
| *b*_L_ [L/g] | 0.0704 | 0.0653 | 0.0666 | 0.0984 | 0.121 |  | 0.0572 | 0.0429 | 0.0514 | 0.0589 | 0.117 |

References

1. Rosés M, Canals I, Allemann H, et al. (1996) Retention of Ionizable Compounds on HPLC. 2. Effect of pH, Ionic Strength, and Mobile Phase Composition on the Retention of Weak Acids. Anal Chem 68:4094–4100. doi: 10.1021/ac960105d

2. Bosch E, Espinosa S, Rosés M (1998) Retention of ionizable compounds on high-performance liquid chromatography: III. Variation of pK values of acids and pH values of buffers in acetonitrile–water mobile phases. Journal of Chromatography A 824:137–146. doi: 10.1016/S0021-9673(98)00647-5

3. Kielland J (1937) Individual Activity Coefficients of Ions in Aqueous Solutions. J Am Chem Soc 59:1675–1678. doi: 10.1021/ja01288a032

4. Gagliardi LG, Castells CB, Ràfols C, et al. (2007) Static Dielectric Constants of Acetonitrile/Water Mixtures at Different Temperatures and Debye−Hückel A and a0B Parameters for Activity Coefficients. J Chem Eng Data 52:1103–1107. doi: 10.1021/je700055p

5. Albright PS, Gosting LJ (1946) Dielectric Constants of the Methanol-Water System from 5 to 55°1. J Am Chem Soc 68:1061–1063. doi: 10.1021/ja01210a043

6. Baciocchi R, Juza M, Classen J, et al. (2004) Determination of the Dimerization Equilibrium Constants of Omeprazole and Pirkle’s Alcohol through Optical-Rotation Measurements. Helv Chim Acta 87:1917–1926. doi: 10.1002/hlca.200490172
